# A history of International Headache Society grants and their impact on headache careers

**DOI:** 10.1177/03331024221107384

**Published:** 2022-06-13

**Authors:** Francesca Puledda, Shuu-Jiun Wang, Hans-Christoph Diener, Henrik Winther Schytz

**Affiliations:** 1 Headache Group, Wolfson CARD, Institute of Psychiatry, Psychology and Neuroscience, King's College London, London, United Kingdom; 2 Neurological Institute, Taipei Veterans General Hospital, Taipei, Taiwan; 3 College of Medicine, National Yang Ming Chiao Tung University, Taipei, Taiwan; 4 Medical Faculty of the University Duisburg-Essen, Essen, Germany; 5 Danish Headache Center, Department of Neurology, Rigshospitalet-Glostrup, Faculty of Health and Medical Sciences, University of Copenhagen, Copenhagen, Denmark

**Keywords:** Headache, migraine, International Headache Society, fellowship, grant, funding

## Abstract

**Background:**

The International Headache Society has been offering multiple award opportunities for young researchers and clinicians for many years, with the aim of supporting the development of careers in headache science and medicine.

**Methods:**

In order to assess the outcomes of the International Headache Society award grants, a questionnaire was sent to all previous recipients, investigating a series of aspects related to their work, both during and after award completion.

**Results:**

Of 44 total questionnaires sent, 36 were returned. Eighty-one percent of the recipients reported to have remained in the headache field since the award, half of them held a current academic position and over three-quarters had stayed in contact with the host institution. The totality of questionnaire responders stated that the grant had had a significantly positive impact on their careers.

**Conclusions:**

The International Headache Society grants have assisted many young researchers in building an academic and clinical career in the field of headache, throughout the years.

## Introduction

One of the leading purposes of the International Headache Society (IHS) is to advance headache science and education worldwide. Over the years, the IHS has created and funded a number of training and grant programs, which have been directed at young trainees who are in the process of developing a career in headache research and clinical practice. These awards have typically been undertaken by young investigators who have traveled to large centers with a strong track record of clinical knowledge and research in headache. Different schemes are in place in order to fulfil different purposes, with some awards being directed to clinical and basic scientists alike, and others being offered to neurology trainees or neurologists in order to enhance their clinical skills in headache.

The *IHS Fellowship* is a one-year award for a total of £50,000, which was first started in 2011. The scope of the fellowship is to support impactful research from young investigators who are within six years of completing their training (either an MD, a PhD, or medical specialty training). This grant scheme is open to applicants from both a basic or clinical background.

The *Trainee/Pioneer Program* enables young neurologists or neurology trainees from low, lower-middle and upper-middle income countries as defined by the World Bank (1) to visit an international headache center for up to 12 weeks. The award, which totals $10,000 per recipient, has the scope to actively increase knowledge on headache disorders and specialized headache management. It is also intended to spread this knowledge to the recipients’ home countries, for the ultimate benefit of local headache patients.

The *Short-Stay Scholarship* is a clinical training period of up to 6 weeks at an international headache center, which was first awarded in 2013. In years corresponding with the International Headache Congress (IHC), the program is held in the same region as the congress, and winning scholars are also funded to attend this. When taking place in alternate years with respect to IHC, the scholars receive funding to attend a regional headache conference. Funding for this scheme has been €5,000 up to 2016 and €6,000 from 2017. 

Finally, the *Allied Specialties Scholarship*, which was pioneered in 2021, facilitates clinical training or a research project through a scholarship directed at specialists in fields that are allied to headache medicine, such as nurses and physiotherapists.

## Methods

With the aim of assessing the outcomes of the different grant schemes, a structured questionnaire was sent via email on behalf of the IHS Education Committee, Science and Research Committee, and Junior’s Group, to all awardees of the above-mentioned grants (Fellowship, Trainee Program and Short-Stay Scholarship). All questionnaire recipients had obtained a grant between 2011 to 2020 and all had been actively completed.

The questionnaire asked for the recipient’s personal details (name, nationality, current position and institution, email address) and details of the grant (dates, host institution name and mentor, title of study). It also included six yes/no questions aimed at understanding the impact the grant had on the recipients’ careers and their ongoing presence in the headache field. These were: 1) Are you still working in the headache/neurology field?; 2) Are you a member of your national headache society?; 3) Are you a member of any IHS or national society Committees/Special Interest Groups?; 4) Have you published any peer-review articles that were related to your stay?; 5) Are you still in contact and collaborating with the headache center you visited?; 6) How have you benefited from the IHS grant? If the recipient answered yes, they were further asked to elaborate details in their answer.

## Results

The turnover for questionnaire completion was high, over 80% in all three groups and the main results of the questionnaire assessment have been summarized in Table 1.

**Table 1.** Results from the questionnaire. Table shows total numbers and in parenthesis % of completers.

	Fellowship	Trainee program	Short-stay scholarship	Total
Total awarded	20	12	24	56
Total questionnaire recipients	15	10	19	44
Total completed	13 (87)	8 (80)	15 (79)	36 (82)
Currently in academic position	10 (77)	3 (38)	6 (40)	19 (53)
Currently actively working in headache	8 (62)	8 (100)	13 (87)	29 (81)
Currently member of national headache society	4 (33)	7 (88)	13 (87)	24 (69)
Currently member of IHS or national society committees	4 (33)	5 (63)	9 (60)	18 (51)
1st author publications from fellowship	10 (83)	4 (50)	5 (33)	19 (54)
Currently in contact or collaborating with host institution	11 (85)	6 (75)	11 (73)	28 (78)
Positively benefitted from grant	13 (100)	8 (100)	15 (100)	36 (100)

**Figure 1.**
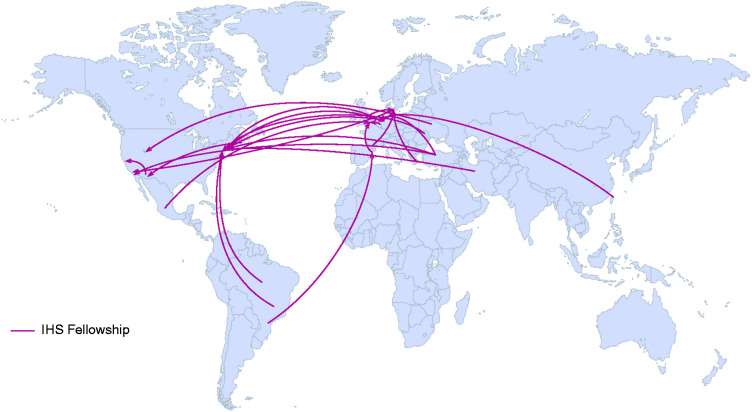
World map showing country of origin and final destination (i.e. host institution) of the twenty recipients of the IHS fellowship, from its start in 2011 to the present day.

With regards to IHS Fellowships, of the total 20 awarded since the start of the scheme, five are currently ongoing. The remaining 15 recipients were sent the questionnaires and 13 completed it (one recipient was not contactable, but information regarding the grant outcome was delivered by a former mentor). Of these, currently 77% have obtained an academic position, which in over half the cases involves headache. Ten former IHS fellows have published at least one first author paper in collaboration with the host institution, and several of these publications have been accepted in high impact journals (2–11). Figure 1 shows the country of origin and of destination of all recipients of the IHS Fellowship.

The Trainee Program has been awarded to 12 people, and two of these awards are currently ongoing. Eight of the remaining ten recipients who completed this scheme responded to the questionnaire. Of these, all of them have remained active in the headache field, with at least five running independent headache clinics in their country of origin. The large majority of former trainees have been participating in the activities of the IHS or their local headache society in the country of origin, when these exist. Even if this training scheme is mostly clinical in nature and shorter than a fellowship, relevant first author publications have arisen from it as well (12,13).

A total of 24 Short-Stay Scholarships have been awarded over the years; five of these were originally awarded in 2020 and had to be rescheduled for the current year due to the COVID-19 pandemic. Of the 19 recipients who were contacted, 15 completed the questionnaire. A high proportion of them are still active in the headache field and the large majority has kept some form of contact with the host institution, usually in the context of discussions of complex clinical cases or for scientific collaborations. Importantly, two scholars have been instrumental in setting up a national headache society in their home country, upon returning from the program.

Finally, two Allied Specialties Scholarships were awarded in 2021 but deferred to 2022 due to the COVID-19 pandemic. Therefore, questionnaires were not sent out to these recipients.

**Figure 2.**
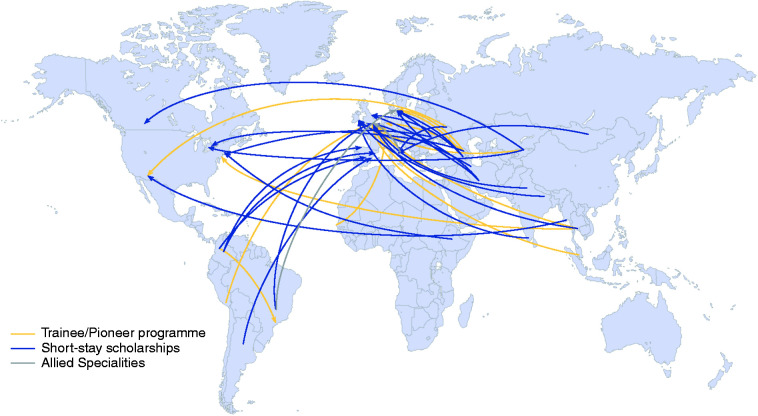
World map showing country of origin and final destination (i.e. host institution) of the recipients of the trainee programme (yellow line), the short stay scholarship (blue line) and allied specialties programme (grey line).

Figure 2 shows the origin and destination of recipients for the Trainee Program, Short-Stay Scholarships and Allied Specialties Scholarships, and Table 2 summarizes the countries of origin and destination for the totality of awards.

**Table 2.** Countries of origin and destination for all grants, per year of award.

Type of award	Year awarded	City and country of origin	Institution and country of destination
Fellowship	2021	Curitiba, Brazil	Vall d'Hebron Institute of Research, Barcelona, Spain
		Phoenix, AZ, USA	Stanford University, Stanford, CA, USA
		Toulouse, France	Danish Headache Center, Glostrup, Denmark
	2020	Athens, Greece	Danish Headache Center, Glostrup, Denmark
		Queretaro, Mexico	King’s College London, UK
	2019	Leiden, Netherlands	Massachusetts General Hospital, Harvard Medical School, Boston, MA, USA
		Birmingham, UK	Danish Headache Center, Glostrup, Denmark
	2018	Liege, Belgium	King’s College London, UK
		Ankara, Turkey	Leiden University Medical Center, Netherlands
	2017	Leiden, Netherlands	Massachusetts General Hospital, Harvard Medical School, Boston, MA, USA
		Taipei, Taiwan	Universitäts-Krankenhaus Eppendorf (UKE), Hamburg, Germany
	2016	Ankara, Turkey	Massachusetts General Hospital, Harvard Medical School, Boston, MA, USA
	2015	Glostrup, Denmark	Erasmus Medical Center, Rotterdam, Netherlands
	2014	Barcelona, Spain	King’s College London, UK
	2013	Ankara, Turkey	UCSD, San Diego, CA, USA
	2012	Tehran, Iran	Massachusetts General Hospital, Harvard Medical School, Boston, MA, USA
	2012	Leiden, Netherlands	Massachusetts General Hospital, Harvard Medical School, Boston, MA, USA
	2012	Halifax, Canada	University of Arizona and Mayo Clinic, AZ, USA
	2011	Glostrup, Denmark	UCLA School of Medicine, Los Angeles, CA, USA
	2011	Kyiv, Ukraine	University of Utah, Salt Lake City, UT, USA
Trainee Program	2020	Medellin, Colombia	São Paulo Headache Center, Sao Paolo, Brazil
		Bangkruai, Thailand	King's College London, UK
		Serdang, Malaysia	King's College London, UK
	2019	Tbilisi, Georgia	Danish Headache Centre, Glostrup, Denmark
	2018	Tbilisi, Georgia	University of Duisburg-Essen, Essen, Germany
		Cairo, Egypt	King’s College London, UK
	2017	Arequipa, Peru	King’s College London, UK
	2016	Kharkiv, Ukraine	Mayo Clinic, Scottsdale, AZ, USA
	2015	Bishkek, Kygrystan	King’s College London, UK
	2014	Kutaisi, Georgia	Danish Headache Centre, Glostrup, Denmark
	2014	Bangkok, Thailand	Jefferson Headache Centre, Philadelphia, PA, USA
	2013	Dakar, Senegal	CHR Citadelle, Liege, Belgium
Short-Stay Scholarship	2020	Ribeirao Preto, Brazil	Guy's and St Thomas, London, UK
		Isfahan, Iran	Danish Headache Center, Glostrup, Denmark
		Kazan, Russia	Danish Headache Center, Glostrup, Denmark
		Zanjan, Iran	King’s College London, UK
		Yereva, Armenia	Danish Headache Center, Glostrup, Denmark/Hull Royal Infirmary, Hull, UK
	2019	Patna, India	Tallaght Hospital, Dublin, Ireland
		Coimbatore, India	Beaumont Hospital, Dublin, Ireland
		Belgrade, Serbia	Mater Misericordiae University Hospital, Dublin, Ireland
	2018	Bogota, Colombia	Vall d'Hebron University Hospital, Barcelona, Spain
		Ulaanbaatar, Mongolia	Pediatric Hospital Bambino Gesu, Rome, Italy
		Kharkiv, Ukraine	Pediatric Hospital Bambino Gesu, Rome, Italy
	2017	Bishkek, Kygrystan	University of Western Ontario, London, Canada
		Tbilisi, Georgia	Hamilton Canadian Headache Observership, Toronto, Canada
		Bishkek, Kygrystan	University of Calgary, Canada
	2016	New Deilhi, India	National Hospitals for Neurology and Neurosurgery, London, UK
		Isfahan, Iran	Department of Neurology, Hull Royal Infirmary, Hull, UK
		Bangkok, Thailand	King’s College London & Pain and Neuromodulation Centre, London, UK
	2015	Cordoba, Argentina	Hospital Clínico Universitario, Valencia, Spain
		Medellin, Colombia	Hospital Ruber Internacional, Madrid, Spain
		Bogota, Colombia	University Hospital Central de Asturias, Oviedo, Spain
	2014	Chisinau, Moldova	Danish Headache Centre, Glostrup, Denmark
	2013	Yangon, Myanmar	Mayo Clinic, Scottsdale, AZ, USA
		Chisinau, Moldova	Albert Einstein College of Medicine, New York, NY, USA
		Addis Ababa, Ethiopia	Stanford Headache Program, Stanford, CA, USA

Considering all grants schemes together, a total of 29 awarded recipients (81% of questionnaire completers) have remained in the headache field, however, this was much higher for the trainee program (100%) than for the fellowship program (62%). Looking at individual cases, the main reason for former fellows leaving the field was due to the difficulty in pursuing a scientific career in academia altogether, particularly in the case of those with a basic science background. In the case of clinicians, the headache field was mostly abandoned while undertaking a neurology residency program.

The majority of grant recipients have remained in contact or in active collaboration with their host institution; two have since stayed in the same laboratory/research group following the end of the award period, and another three have stayed in the country where the fellowship took place for further career opportunities.

Importantly, all the completers of the questionnaire, regardless of the type of award received, judged it to have positively impacted and shaped their careers. The awards were explained to have helped them in several ways through their careers, from increasing knowledge to allowing their growth as scientists and researchers. By opening further funding and collaboration opportunities, the grants were described as having directly determined the work of the awardees, their current lines of research as well as relevant academic and scientific positions obtained after the grant. In several cases, the grant was even instrumental in shaping life choices such as changing country of residence after the end of the award.

A limitation of our study is certainly the small sample size due to the fact that the awards are limited in number because of monetary resources, and have started relatively recently. Further, their duration can be long, with several recipients currently involved in a grant and being thus unable to take part in the questionnaire. Repeating this study in a few years might help address these issues and provide further information on the long-term effects of the awards. A further source of bias resides in the fact that the awardees who benefitted the most might have been more inclined to respond. Finally, it is important to acknowledge that, although these schemes have benefitted people from a large variety of countries, they cannot fully counteract the inequality in opportunities between different regions of the world.

In conclusion, this short questionnaire survey helped to consolidate the importance of grant schemes created by the IHS in order to promote the careers of young scientists and clinicians in the field of headache. It also shows that improvement needs to be made for future opportunities, particularly in facilitating basic scientists and clinicians who struggle to remain in the academic field. Some possibilities could include the addition of targeted training programs focused on academic development, the creation of specific networking events to connect past awardees with newly awarded recipients, and finally a continuation of support from the host institutions to former grant holders through virtual mentoring programs.

## Article highlights

- The IHS grants have been instrumental in allowing young neurologists and researchers in building an academic and clinical career in the field of headache, throughout the years.
